# Towards Intelligent Data Analytics: A Case Study in Driver Cognitive Load Classification

**DOI:** 10.3390/brainsci10080526

**Published:** 2020-08-06

**Authors:** Shaibal Barua, Mobyen Uddin Ahmed, Shahina Begum

**Affiliations:** School of Innovation, Design and Engineering, Mälardalen University, Högskoleplan 1, 72220 Västerås, Sweden; mobyen.ahmed@mdh.se (M.U.A.); shahina.begum@mdh.se (S.B.)

**Keywords:** cognitive load, machine learning, multimodal data analytics, multicomponent signals

## Abstract

One debatable issue in traffic safety research is that the cognitive load by secondary tasks reduces primary task performance, i.e., driving. In this paper, the study adopted a version of the n-back task as a cognitively loading secondary task on the primary task, i.e., driving; where drivers drove in three different simulated driving scenarios. This paper has taken a multimodal approach to perform ‘intelligent multivariate data analytics’ based on machine learning (ML). Here, the k-nearest neighbour (k-NN), support vector machine (SVM), and random forest (RF) are used for driver cognitive load classification. Moreover, physiological measures have proven to be sophisticated in cognitive load identification, yet it suffers from confounding factors and noise. Therefore, this work uses multi-component signals, i.e., physiological measures and vehicular features to overcome that problem. Both multiclass and binary classifications have been performed to distinguish normal driving from cognitive load tasks. To identify the optimal feature set, two feature selection algorithms, i.e., sequential forward floating selection (SFFS) and random forest have been applied where out of 323 features, a subset of 42 features has been selected as the best feature subset. For the classification, RF has shown better performance with *F*_1_-score of 0.75 and 0.80 than two other algorithms. Moreover, the result shows that using multicomponent features classifiers could classify better than using features from a single source.

## 1. Introduction

Driving a vehicle requires dynamic adjustment of cognitive control, here, both visual and physical tasks are crucial to keep the driving performance to an acceptable level within a comfortable effort [1]. While driving a vehicle, drivers are often occupied with many other activities such as using a mobile phone, listening to the radio, or having a conversation with a passenger, etc. Moreover, new advanced in-vehicle information systems embedded in the modern vehicles could create distracted driving scenarios and may affect the driving performance [2,3,4]. Thus, these secondary activities, i.e., activities not related to driving require extra cognitive processes in ways that the driver can still keep their eyes on the road and hands on the steering wheel while being involved in other activities at the same time, and this refers to the ‘cognitive load activities’. It is reported that more than 90% of traffic crashes are assigned to the driver’s error, whereas 41% of them are due to inattention, distraction, and cognitive load activities [5]. Further, the risk concerning traffic safety and driving performance anticipating cognitive load activities have been addressed in [6,7].

Many studies have been pursued to understand the consequences of secondary task or dual-task demands while driving and different types of data such as physiological, driving behavioural, and subjective measures have been used to evaluate the driver’s mental effort [1,8,9,10]. In this paper, the attention selection model (ASM) [11], based on the n-back task has been employed to impose the cognitively loading secondary task while driving. The ASM is a conceptual model of attention selection and multitasking in everyday natural driving situations. The n-back task is a continuous performance task commonly used as an assessment of working memory load [12,13]. The n-back task can vary on the task difficulty or complexity, such as very mild task demand (0-back), moderate task demand (1-back), and a high level of task demand (2-back) [14]. Since it is difficult to characterise the task demand from the invested effort on the secondary task by the drivers [1], the alternative is to use physiological measures and this work aims to explore the classification of cognitive load activities i.e., the n-back task events.

Several physiological signals such as electroencephalography (EEG), electrooculography (EOG), electrocardiogram (ECG), heart rate (HR), and heart rate variability (HRV) (the variation in the beat to beat intervals of the heart), galvanic skin response (GSR), and respiration rate (RR) signal have become object measures of cognitive load. Several studies have also considered the variations in drivers’ behavioural data obtained from vehicular signals for drivers’ cognitive load classification [15,16]. Machine learning methods that have been used for detecting the driver state, e.g., cognitive load include SVM [17,18,19,20,21,22,23,24,25], artificial neural network [2,26,27,28,29], random forest [30,31], deep learning [32,33], and case-based reasoning [34,35,36,37]. The performance of cognitive load classification is often poor when there are uncertainties—such as participants failing to perform some task or, in a real-time system. The accuracy could be improved, for example, using a suitable window size, which influences the delay that occurs between the onset of a cognitive load task and when changes are detected in the driver’s performance due to the higher cognitive load [2,21]. Several studies use multimodal data in machine learning models to classify driver states [2,38,39].

This paper aims for intelligent data analytics using ML to discriminate the cognitive load task from normal driving in different driving situations such as visual cue in traffic, incoming traffic, and traffic environment. The cognitive load task classification based on physiological measures have become practical and they have shown much potential because of their granularity and high degree of responsiveness. However, in many occasions physiological measures suffer from both confounding factors and noise [40]. Hence, this paper focuses on a multimodal approach based on multicomponent signals to measure the cognitive load and to classify different cognitive load tasks. Here, several ML algorithms, e.g., k-nearest neighbour (k-NN), support vector machine (SVM), and random forest (RF) are applied for the classification. Again, two feature selection algorithms, i.e., sequential forward floating selection (SFFS) and random forest with mean decrease accuracy (MDA) are used to identify an optimal feature set. In this paper, physiological signals, i.e., EEG, EOG, GSR, ECG, and RR are fused as a driver behavioural data and combined with driving context, i.e., driving conditions in the scenarios (see Section 2). Again, a number of vehicular features, e.g., lateral speed, steering wheel angle (SWA), yaw and yaw rate, and lateral position are also being considered. Considering the study design, both multiclass and binary classification have been performed, where classifiers are trained using 5-fold cross-validation on the training datasets and finally, evaluated with the test datasets.

## 2. Materials and Methods

### 2.1. Study Design and Data Set

**Figure 1:**
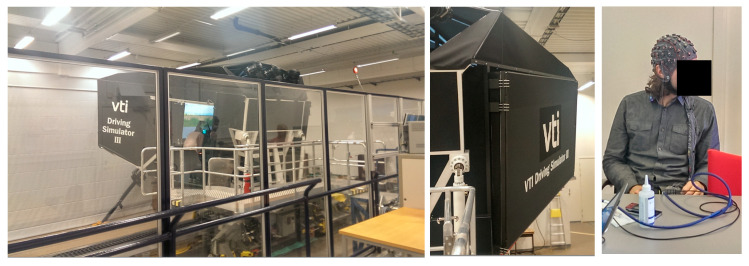
VTI simulator III and electroencephalography (EEG) electrodes setup on a participant.

The experimental study took place at the Swedish National Road and Transport Research Institute (VTI), Linköping, Sweden, using a high-fidelity moving-base driving simulator (VTI Driving Simulator III (https://www.vti.se/en/research-areas/vtis-driving-simulators/)), see Figure 1. The study was approved by the regional ethics committee at Linköping University, (Dnr 2014/309-31) and each participant signed an informed consent form. The simulator was a car cabin consisting of front seats of a SAAB 9-3 with automatic transmission. It could simulate the movements and forces by moving, rotating, or tilting the part of the simulator with projector screens. A vibration table enables the simulation of the road surface contact. It had three liquid-crystal display systems for rear mirrors and six projectors for visualization of the frontal view with a horizontal field of view of 120 degrees. The study that collected the cognitive load dataset consisted of two test series that contained recordings from 66 participants (33 in test series 1 and 33 in test series 2). All the participants were male with no known diseases or medications, aged between 35 and 50 (42.47 ± 4.39 years), and had held a valid driver’s license for more than ten years. To obtain homogeneity, only males were chosen with the aforementioned criteria. Further, participants were not professional drivers (e.g., taxi and heavy vehicle driver), no extremes in terms of self-reported personalities (extrovert or introvert), and self-reported normal sensitivity to stressful situations. To assess stress tolerance, each participant had to fill up a questionnaire after the end of each driving session. The self-reported questionnaire used a scale of 0–6, where ‘0’ means low-stress tolerance and ‘6’ means the high-stress tolerance; whereas for anxiety ‘0’ indicates low and ‘6’ indicates high anxiety. However, the personality and stress sensitivity have not been taken into consideration in this paper.

**Figure 2:**
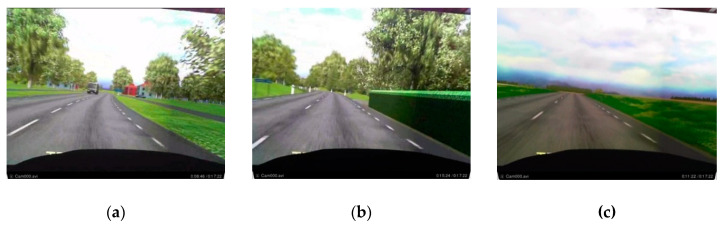
Three of the study scenarios, (**a**): Car approaching the crossing from the right (CR); (**b**): Hidden exit on the right side of the road with a warning sign (HE); and (**c**): Strong side wind in open terrain (SW) scenario.

The driving environment in the simulator consisted of three recurring scenarios in which the simulated road was a rural road with one lane in each direction, some curves and slopes, and a speed limit of 80 km/h. The three scenarios were (1) four-way crossing with an incoming bus and a car approaching the crossing from the right (CR), (2) a hidden exit on the right side of the road with a warning sign (HE), and (3) a strong side wind in open terrain (SW). Figure 2 represents examples of these study scenarios. In Figure 2, the image in the left shows the CR scenario, the middle one shows the HE scenario, and the rightmost image shows the SW scenario. Thus, these scenarios implied threats in off-path locations without requiring the drivers to change their responses. As a within-measure study, each scenario was repeated four times during the approximately 40 min driving session where the participants were involved either in a cognitive load task, i.e., a 1-back or 2-back task, or were driving to pass a scenario (baseline or no-task). In the first test series, participants performed the normal driving and 1-back task while driving. However, in the second test series, the participants performed all three task conditions in the hidden exit and four-way crossing scenarios. The no-task and 2-back tasks were only performed under the side wind in the open field scenario. The 1-back and 2-back tasks are considered as the secondary auditory tasks, where a number is orally presented through the simulator’s speakers at an interval of 2 s. The participants had to respond whenever the last presented number was the same as the previous one (1-back) or two steps earlier (2-back).

The physiological signals were acquired using a multi-channel amplifier with active electrodes (g.HIamp, g.tec Medical Engineering GmbH, Austria). The electroencephalography (EEG) electrodes were positioned based on the 10–20 system providing a 30-channel recording. The EEG signals were band-pass filtered between 0.5 and 60 Hz using an 8th order Butterworth filter, and frequencies between 48 and 52 Hz were removed using a 4th order Butterworth notch filter. In addition, electrooculography (EOG) (horizontal with electrodes at the outer canthi and vertical with electrodes above/below the left eye) were also acquired. ECG was measured using disposable ECG electrodes with a snap connection to the wiring. The respiration rate (RR) was measured using a SleepSence chest strap which was connected to the upper body. The skin conductance was measured using reusable gold-plated cup electrodes with conductive cream; the electrodes were connected to a GSR sensor (g.tec g.GSRsensor). Vehicular parameters such as lateral position (LatPos), lateral speed (LatSpeed), steering wheel angle (SWA), land departure (LanDep), and yaw rate were recorded in the simulator control computer.

### 2.2. Classification Approach

**Figure 3:**
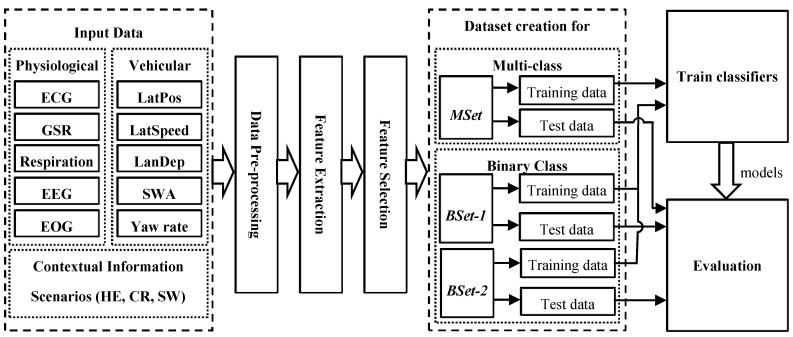
Block diagram of the steps for driver cognitive load classification using multivariate data.

The aim of the classification task was to differentiate the driving events with the cognitive load task from normal driving. The influence of the scenarios on classification is evaluated by classifying cognitive load tasks for all individual situations. Each scenario had a duration of 60 s where the first 10 s of the data were discarded to adjust the stability of the driver with the cognitive load task. Hence, a 50 s recording of each scenario was used for feature extraction. Figure 3 shows the overall schematic diagram of the classification task. The steps include data gathering, data pre-processing, feature extraction, feature selection, dataset creation, training classifiers, and finally evaluation of each of the classifiers using the test dataset. Here, the data were gathered through a study with 66 participants (33 in test series 1 and 33 in test series 2) presented in Section 2.1.

#### 2.2.1. Data Pre-Processing

The driving task involves activities such as looking at the side and rear-view mirror, shifting gear, and changing body position that naturally causes muscle and ocular artifacts in the EEG signals. Therefore, it requires cleaning the EEG signal before extracting frequency component features from the EEG signal. Hence, EEG signals were artifacts handled using an in-house developed tool called ARTE (Automated aRTifacts handling in EEG) [41]. The median filter was used to handle noise in the vehicular data, respiration, and GSR signals. The median filter is particularly useful for removing spiky noise and can separate peaks from a slowly changing signal disturbed by unknown noise distribution [42]. A QRS detection algorithm proposed by [43,44] was used to extract inter-beat-interval (IBI) data from the ECG signal. The obtained IBI data were filtered using the ARTiiFACT tool [45]. The collected raw dataset was in the European data format, which was converted into MATLAB (Matlab 2017b version. https://se.mathworks.com/products/new_products/release2017b.html) data format and all the works were done in MATLAB 2017b version.

#### 2.2.2. Feature Extraction

Various features are extracted from both the physiological and vehicular parameters as presented in Table 1. Here, the feature vector consists of 323 extracted features with total observations of 721, where 306 of them are baseline or no-task, 237 observations are the 1-back task, and 178 observations are the 2-back task.

EEG Features: From each of the 30 channel EEG signal power, the spectral density (PSD) of the δ (<4 Hz), θ (4–7 Hz), α (8–12 Hz), β (12–30 Hz), and γ (31–50 Hz) frequency bands are extracted as features. The Welch’s method [46] is used with 50% overlapping with the Blackman window function. In addition, four different ratios of the PSDs, (θ+α)/β, α/β, (θ+α)/(α+β), and θ/β [47], are also estimated as features. These four ratios indicate the change of slow wave to fast wave of EEG activities over time. According to [47], an increase in the ratio is a good indicator of EEG activity compared to *α* and *θ* alone. Moreover, the authors found that *α* and *θ*, combined with the ratios, could better assess the fatigue condition of the drivers. Hence, nine features from each EEG channel resulted in 270 EEG features for each 50 s time segment driving event. The motivation of using EEG is that as the cognitive load increases, changes in alpha and theta powers in EEG have been observed in various studies [48,49,50]. It is reported that alpha and theta powers increase as the cognitive load increases [48,49,50]. Another common approach in the Brain-Computer interface is to apply the independent component analysis (ICA) to extract features from the PSDs of ICA components [51,52]. EEG classifications for different mental workload activities have been performed in [53,54]. However, depending on the study design and the type of cognitive load under scrutiny, the results are often ambiguous [48,50].

EOG Features: The EOG features derived from the vertical EOG using an automatic blink detection algorithm based on derivatives and thresholding was developed by Jammes and Sharabty [55]. The average spontaneous eye blink rate of a person is 15–20 per min [56]. The eye blink frequency increases as the cognitive load increases [48,57,58], whereas the decrease in blink duration is observed by [59].

ECG Features: Heart rate (HR) and heart rate variability (HRV), i.e., measure of the variations in time between each heartbeat, are two measures that can vary with the increasing cognitive load. HRV measures beat-to-beat (R–R interval) variations in terms of consecutive heartbeats articulated in the normal sinus rhythm from electrocardiogram (ECG) recordings [60,61]. HR and HRV features are obtained from the pre-processed interbeat interval (IBI) data. In time domain, statistical methods are applied to extract the time domain features. To obtain frequency domain features, the IBI data are transformed via FFT transformation. The PSDs of low frequency (LF) (0.04 to 0.15 Hz) and high frequency (HF) (0.15 to 0.40 Hz), LF/HR ratio, and total power are estimated. The time and frequency domain measures quantify the variability of the heart rate fluctuation characteristic in time scales. On the other hand, the non-linear measures quantify the structure or complexity of the R-R intervals, i.e., IBI data. Non-linear measures such as detrended fluctuation analysis, sample entropy, approximate entropy, and permutation entropy methods were applied to extract complexity from the IBI data [62]. An increased HR with respect to the increasing cognitive load has been reported in several studies; in contrast, the time domain measures of HRV such as mean RR, SDNN, RMSDD, pNN50, and HF power band (0.15–0.50 Hz) of HRV in the frequency domain decrease [14,63,64]. An increase in the LF power (0.04–0.15 Hz) and the LF/FH ratio of HRV have been associated with higher mental workloads [64,65,66].

GSR Features: GSR measures the electrical conductivity of the skin and can provide changes in the human sympathetic nervous system [67]. GSR is significantly correlated with the cognitive load task demand and usually used for the level of cognitive load classification [40,67,68]. In time domain several estimations, i.e., number of peaks, the amplitude of the peaks (maxima-minima), duration of the rise time of each peak, index of the detected peaks in the GSR signal, mean value, standard deviation, first quartile value, third quartile value, slope value between peak and valley are extracted as features [67]. One feature which is the average power of the signal under 1 Hz is extracted in frequency domain. A comprehensive review of GSR signal interpretation can be found in [69]. Further, relations between cognitive load and GSR features have been discussed in several studies [68,70,71].

Respiration Features: From the respiration rate (RR) signal, arithmetic mean, standard deviation, and kurtosis are calculated as features in time domain. Similar to EEG, Welch’s method has been used to estimate the PSDs from the frequency ranges [0, 0.1], [0.1, 0.2], [0.2, 0.3], [0.3, 0.4], [0.4, 0.7], and [0.7, 1] [72,73]. The cognitive load has a distinct effect on the respiratory behaviour that can differ in sensitivity in the parameters obtained from respiratory signals [74]. According to Hidalgo-Muñoz et al. [72] significant increases in the respiration rate are observed while driving in comparison to the base line condition. Moreover, the RR showed variations with a different level task difficulty and RR accelerated with an increasing cognitive workload.

Vehicular Features: The standard deviation from five time series data namely, lateral speed, steering wheel angle (SWA), yaw and yaw rate [75], and lateral position [15], are extracted as features. The steering wheel reversal rate (SWRR) [15], is defined as the absolute difference between maximum and minimum of the SWA signal. The SWRR is the number of reversals in a time period. Firstly, the raw SWA is smoothed using the Lowess method where the linear model is used for local fitting [76]. In this case, 110 points have been used for the moving average in the linear model. Steering wheel entropy [77,78], high frequency component (0.3 Hz), and number of zero crossings are the other features that are obtained from the SWA signal. Lanex or the fraction of lane exit feature is extracted from the lane departure signal which indicates the driver’s tendency to exit the driving lane. Lanex is defined as the fraction of a given time interval spent outside driving [75]. In several studies, drivers’ behavioural data in relation to vehicular signals such as speed, lateral position, steering wheel angle, etc. have been used to detect and classify drivers’ cognitive load [15,16]. For example, driving performance relies on a right speed [79]. A reduced speed as a compensatory action due to the increased cognitive load is more often used as an indication of behaviour adaption rather than a change in driving performance [80,81]. Östlund and Nilsson [82] presented a few other parameters such as lateral position and steering wheel reversal rate that contribute to the driver’s cognitive load. Wilschut [82] used the steering wheel angle and lane positioning to measure the driving performance. A lane change task can be used to investigate the effects of cognitive load on driving performance [83].

#### 2.2.3. Feature Selection

Feature selection is conducted only on the EEG signals since 270 EEG features are extracted from the 30 channels and many of them were neighbouring electrodes. Some overlapping and redundant features might exist. Hence, sequential forward floating selection (SFFS) [84,85,86] was used to also investigate the intra-feature relationships. SFFS is a successor of the sequential forward selection (SFS) method, which does not suffer from the ‘nesting effect’, and is computationally more efficient than other branch and bound methods [86]. SFFS was wrapped with an SVM classifier to obtain an optimal feature subset. Further, the SVM classification was evaluated using 5-fold cross-validation. For other features, random forest with the mean decrease accuracy (MDA) [87] approach was used in the feature selection process. The idea of using MDA is to find the direct impact of each feature on the performance of the random forest model. Here, a permutation of each feature measures the decreasing accuracy of the model and for the unimportant features the permutation has little effect on the model accuracy. On the other hand, removing important features should drastically decrease the accuracy.

#### 2.2.4. Cognitive Load Classification

For cognitive load classification, data from both the test series are combined, and both multi-class (*MSet* data) and binary class (*BSet* data) classification are defined based on the n-back task and normal driving events. The binary class is defined as the task group and baseline group. For the binary classification, two data sets are created such that the first set (*BSet-1*) baseline consists of normal driving and 1-back task, and the task group contains data of 2-back task. In the second set (*BSet-2*), the baseline includes data from normal driving only, and the task group consists of data from both 1-back and 2-back tasks. These two binary datasets preparation was motivated by the assumption that the 1-back task did not have much influence on the driver (e.g., on working memory) compared to the 2-back task [88]. The *MSet*, *BSet-1*, and *BSet-2* datasets are split into training and test datasets, where the training set contains 70% and the test dataset contains 30% of the data sets. Three separate classifiers k-NN, SVM, and RF are developed and trained using 5-fold cross-validation with the training datasets and later evaluated with the test datasets. In addition, the binary classification was performed for both scenario-wise and task-wise to discriminate the effect of scenarios on the cognitive load task. Here, only the training dataset was used in the feature selection step. The training set is further divided into two sets, where 80% of the training data is used for SFFS and MDA, and 20% of the training data is used as a validation set.

k-NN is a simple memory-based algorithm that uses the observations in the training set to find the most similar properties of the test dataset [89]. In this work, the Euclidean distance function is used with a ‘squared inverse’ distance weight and K = 5 was considered. SVM finds the hyperplane that not only minimizes the empirical classification error but also maximizes the geometric margin in the classification [90]. SVM can map the original data points from the input space to a high dimensional feature space such that the classification problem becomes simple in this feature space. In this study, an SVM with a Gaussian kernel was used for the classification task. A popular ensemble algorithm in machine learning is RF, that consists of a series of randomizing decision-trees, where the output is the majority vote of all these decision-trees [91]. One important aspect of RF is that it does not assume independence of features. In the driving context, data is often noisy and rarely linearly separable into a different mental state [92]. RF is implemented using bagging, which is the process of bootstrapping the data plus using the aggregate to make a decision. During classification, MATLAB’s *fitcknn* function is used for k-NN, the *fitcecoc* function with an SVM template is used for SVM, and the *fitcensemble* function with 4357 tree splits is used for RF. The three classifiers were evaluated considering confusion matrices, accuracy, balanced accuracy (BACC), Matthews correlation coefficient (MCC), *F*_1_-score, sensitivity, and specificity.

## 3. Results

### 3.1. Feature Selection

**Figure 4:**
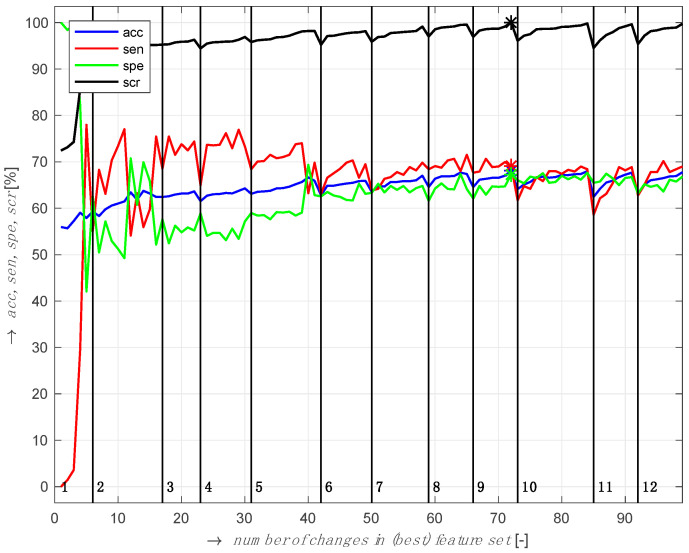
EEG feature selection using sequential forward floating selection (SFFS) on the training dataset, validated using 10-fold cross-validation.

The SFFS selected θ/β, α/β, (θ+α)/β, θ, β, and α features from seven frontal channels namely, FP1, FP2, F7, F4, FPz, FC2, and FC5. The best classification accuracy was 66% with 11 EEG features. Figure 4 shows the accuracy (acc), sensitivity (*sen*), specificity (*spe*), and classification score (*scr*) defined as $2^{\sin\left(\frac{\pi .sen}{2}\right).\sin\left(\frac{\pi .spe}{2}\right)}$ [85].

The feature subset after feature selection using SFFS and MDA is listed in Table 2. In total, from all the signals, 42 features were selected out of 323 features.

### 3.2. Classification Evaluation

**Figure 5:**
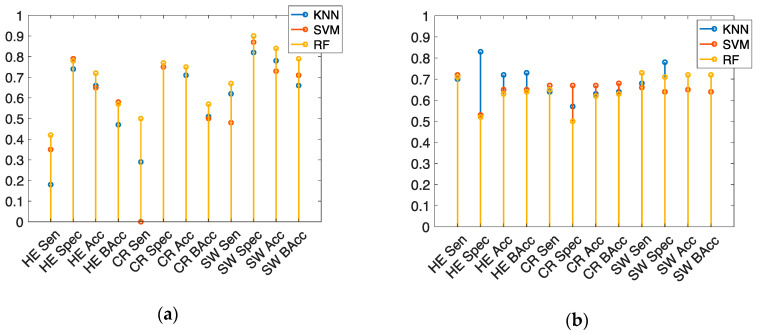
Scenario wise classification performance on the test dataset. (**a**) Shows results for *BSet-1*; and (**b**) Shows results for *BSet-2*.

The scenario wise binary classification was performed to see if there are any effects of the scenarios in the classification performance. Figure 5 shows the performance of binary classification for each scenario using both test datasets of *BSet-1* and *BSet-2*. It can be seen that the balanced accuracies (BAcc) of HE and CR scenarios are lower and higher only for the SW scenario using RF on the data of *BSet-1*. It is important to mention that the ratio of the baseline and task groups in the *BSet-1* is much imbalanced than the *BSet-2*. For both *BSet-1* and *BSet-2*, the BAcc is higher for the side wind scenario. Using the test dataset of *BSet-1*, in the HE scenario, BAcc(s) are 47%, 58%, and 57% for k-NN, SVM, and RF, respectively; in the CR scenario, BAcc(s) are 51% for k-NN, 50% for SVM, and 57% for RF; in the SW scenario, BAcc(s) are 66% for k-NN, 71% for SVM, and 79% for RF.

On the other hand, using the test dataset of *BSet-2*, in the HE scenario, BAcc(s) are 73%, 65%, and 64% for k-NN, SVM, and RF, respectively; in the CR scenario, BAcc(s) are 64% for k-NN, 68% for SVM, and 63% for RF; in the SW scenario, BAcc(s) are 72% for k-NN, 64% for SVM, and 72% for RF.

As observed in Figure 5, the classification may have some influence on the driving scenario, hence a categorical feature is incorporated with the existing features presented in Table 2. Afterwards the multiclass classification was performed using the *MSet* dataset and the binary classification was performed using both *BSet-1* and *BSet-2* datasets.

Multiclass classifications with k-NN, SVM, and RF are performed to investigate how each class contributed to the classification performance. On the training dataset of *MSet*, using 5-fold cross-validation, k-NN achieved 53% classification accuracies, whereas both SVM and RF achieved 59% classification accuracies. Table 3 shows the confusion matrices for the test dataset. RF shows better performance than k-NN and SVM considering the number of correct classifications of each target group.

Table 4 represents the classification summary on the test dataset of *MSet* considering true positive (TP), true negative (TN), false positive (FP), false negative (FN), precision, sensitivity, specificity, and balanced accuracy (BACC). Here, one-vs.-rest was used to determine the target groups in the positive (P) and negative (N) classes. The positive class is the target group that corresponds to either baseline, 1-back, or 2-back task in each column. The negative (N) class consists of the other two target groups, i.e., 1-back + 2-back, baseline + 2-back, and baseline + 1-back. Overall, RF shows better performance considering the balanced accuracy.

Binary classifications were performed using *BSet-1* and *BSet-2*. The observed classification accuracies for k-NN, SVM, and RF with 5-fold cross-validation on the training dataset of *BSet-1* are 79%, 81%, and 82%, respectively. On the training dataset of *BSet-2*, the achieved classification accuracies are 67% for k-NN, 72% for SVM, and 75% for RF. The prediction performance of k-NN, SVM, and RF, on the test dataset of each of *BSet-1* and *BSet-2* is presented in Table 5.

## 4. Discussion

Cognitive loading activities on traffic safety and its relation to driving performance has drawn an increasing attention to the traffic safety research issue. Here, the cognitive load dataset was acquired and analysed to understand the effect of cognitive load on traffic safety. Driving a vehicle is an anticipatory task where a driver needs adaptation concerning the road users’ behaviours and their actions which are dynamic in nature. Driving is often considered as a process that is nearly automated, partially self-paced, and a satisficing task [93]. A driver can somewhat distribute the load of the driving task by deciding when, where, and what they do. This holds true not only for driving-related tasks but also for secondary tasks such as talking on a mobile phone or conversing with a passenger while driving. Most of the time this works well, but sometimes it does not [6,7,94,95,96]. In the cognitive load theory, working memory is considered as an executive function that holds information and mentally processes that information [97]. Hence, in this paper the cognitive load is considered as the amount of cognitive resources (i.e., mechanisms necessary for cognitive control) used at a certain time [11]. The effect of cognitive load on traffic safety is considered utilizing the attention selection model (ASM) [11]. According to the ASM model, the cognitive load does not affect the automatic performance but impairs subtasks that rely on cognitive control.

Among the physiological signals, the EEG is one accessible technique to measure cognitive load and the EEG signal analysis can detect changes in an instantaneous load and the effects of cognitively loading secondary tasks. The EEG feature selection in cognitive load classification showed the best feature subset selected by the SFFS algorithm, containing θ/β, α/β, (θ+α)/β, θ, β, and α features from only the frontal electrode. Features from the frontal region might suggest only motor function, and attention affected the cognitive loading activities. HRV from ECG, GSR, and RR features might be better indicators for cognitive load classification, a finding also supported by other studies. HRV features can be an important indicator for classifying cognitive load because cognitive load modulates the sympathetic and parasympathetic nervous systems inversely to driver sleepiness [98]. The time domain GSR, i.e., the peak amplitude, the duration of the rise time of each peak, and the mean GSR value were found to be useful indicators for cognitive load detection when a person is under the influence of different stress levels [99]. Furthermore, the states depend on the experimental design, driving environment, confounding factors, etc., and hence, multi-variate data and data fusion considering the driving context are needed to accurately assess the cognitive load. It should be noted that subjective measures, for example, the NASA-TLX [100] or the DALI (driving activity load index) [101], require understanding the importance of physiological features and vehicular features.

In this paper, the cognitive load classification was performed based on the baseline (just driving) and n-back task (1-back and 2-back). This approach could have affected the classification performance because the influence of a cognitive loading task (e.g., on working memory) might not be the same for everyone, especially for the 1-back task. It is noteworthy to mention that the cognitive load classification distinguishes among different levels of cognitive-level tasks and does not imply how cognitively loaded participants are performed during the n-back task. In terms of classification, the problem lies in the class noise in the dataset. Apart from the analysis presented in this paper, several other classification experiments [102] have been conducted considering features according to (1) cerebral activities recorded via EEG, (2) cerebral activities recorded via EEG and eye blink waveform via EOG, (3) non-cerebral physiological signals recorded via HRV, GSR, and respiration, and (4) driving behavioural data based on vehicular parameters obtained from the control computer. The results showed poor performance than combining all features as the results presented in this paper. By using only the EEG features from *BSet-1* (i.e., baseline = normal + 1-back task, and the task group = 2-back task) dataset, the height accuracy of 74%, 46% sensitivity, and 78% specificity was obtained by the RF algorithm. The performance was decreased using only the EEG features from the *BSet-2* (i.e., baseline = normal, and the task group = 1-back task + 2-back task) dataset. Again, RF showed the best performance with 57% accuracy, 61% sensitivity, and 49% specificity. When features from both the EEG and EOG signals were combined, a slight improvement was observed in the classification performance using both the *BSet-1* and *BSet-2* datasets. Here, k-NN showed the best performance for both *BSet-1* and *BSet-2* datasets and the accuracy, sensitivity, and specificity were around 75%, 59%, and 81%, respectively. It has been observed that using only the vehicular features classification perform similarly as using the EEG features only. However, a combination of features from non-cerebral physiological signals, i.e., HRV, GSR, and respiration, was found to perform better for the classification compared to using only EEG, vehicular, and a combination of EEG and EOG features. The RF algorithm obtained the best performance using the *BSet-1* dataset considering 78% accuracy, 70% sensitivity, and 82% specificity. Similarly, RF showed the best performance using the *BSet-2* dataset considering 73% accuracy, 75% sensitivity, and 69% specificity. In all the cases, i.e., using only the EEG feature, a combination of features from EEG and EOG, features from vehicular signal, and combination of features from ECG, GSR, and RR signals the obtained classification accuracy was not more than 50%, and the sensitivity and specificity were around 55% and 60%, respectively.

Overall, a 10% improvement in the classification performance was observed by using a combination of all multivariate features compared to the performance observed when using only the features from the EEG signals. A 20% improvement in the classification performance for multiclass classification was observed by using a combination of all multivariate features compared to that observed using only the feature based on the vehicular data. The current classification approach implies that it is not individualised; that is, the response pattern is assumed to be the same for all drivers. The scenario-wise classification shows that there is an effect of driving condition on the cognitive load. Thus, integrating contextual information as features can be beneficial to the classification. However, in this work, it was not fully comprehending the consequence of adding contextual features. The limitation of this approach can be overcome by incorporating subjective measures into the study design and adding a wide range of contextual information.

## 5. Conclusions

The objective of this paper was to provide analytics on multivariate data for driver cognitive load classification. The multiclass classification results portray the difficulty to correctly classify when there are imbalance classes in the dataset, which leads to performing the binary classification. These analytics emphasize the study design with a wide range of contextual information and subjective measure to predict or identify the level of cognitive load during driving. It is also found that multicomponent features could improve the overall classification performance. Another important issue of this study was the imbalanced class in the dataset. Hence, in this study, BACC, MCC, and *F*_1_-score were considered along with accuracy, sensitivity, and specificity. It should be noted that though for some occasions *F*_1_-score, sensitivity, and specificity showed reasonable measures but looking at MCC it is evident that the models tend to bias towards the class with higher observations. Though the inclusion of contextual feature is inconclusive, yet it is believed that contextual information not only can improve the classification performance but also can provide insights when it requires interpretation of the ML model. It is argued that the n-back task is an efficient task to measure the individual working memory capacity [103]. The scenario wise classification with a *BSet-2* could better discriminate between normal driving and n-back task compared to the binary classification with *BSet-1*. It can be concluded from the result of the scenario wise classification that the cognitive load impairs the driving subtask that depends on cognitive control which is also the suggestion by ASM. Although studies [103,104] found more discriminatory EEG activity patterns between the n-back tasks, those studies only considered the n-back task as the main discriminatory factor. However, in this study the n-back task is adapted for ASM that may influence the classification performance and supports the idea of ASM that the automatic performances of the driving task are unaffected by the cognitive load.

## Figures and Tables

**Table 1:** List of features extracted from each signal. # represent features.

Signal	# Features	Extracted Features
EEG	270	Frequency bands: δ (<4 Hz), θ (4–7 Hz), α (8–12 Hz), β (12–30 Hz), γ (31–50 Hz), and the ratio (θ+α)/β, α/β, (θ+α)/(α+β), and θ/β
EOG	9	Start position of blink, blink duration calculated from the start position of blink to the end value of blink, lid closure speed, PCV (peak closing velocity), delay of eye lid reopening, duration at 80%, PERCLOS, blink rate, blink count.
ECG	14	Time: Mean heart rate (meanHR), standard deviation of heart rate (sdHR), standard deviations of normal to normal RR intervals (SDNN), root mean square of successive differences between adjacent NN intervals (RMSSD), number of pairs of successive NN intervals with more than 50 ms (NN50), percentage of NN50 (pNN50).
		Frequency: Low frequency power (0.04–0.15 Hz), high frequency power (0.15–0.4 Hz), total power, LF/HF ratio.
		Non-linear: Alpha value of detrended fluctuation analysis (dfaAlpha), sample entropy (SampEn), approximate entropy (ApEn), and permutation entropy (PeEn).
GSR	10	Time: Number of peaks, the amplitude of the peaks (maxima-minima), duration of the rise time of each peak, index of the detected peaks in the GSR signal, mean value, standard deviation, first quartile value, third quartile value, slope value between peak and valley.
		Frequency: Average power of the signal under 1 Hz.
Respiration rate (RR)	9	Time: Mean value, standard deviation, kurtosis.
		Frequency: Power spectra power between the frequency ranges [0, 0.1], [0.1, 0.2], [0.2, 0.3], [0.3, 0.4], [0.4, 0.7], and [0.7, 1].
Vehicular parameters	11	Standard deviation of lateral position, mean squared error of lateral position.
		Standard deviation of steering wheel angle, steering wheel entropy, steering wheel reversal rate, high frequency component (0.3 Hz), number of zero crossings.
		Lanex or fraction of lane exit from lane departure.
		Standard deviation of lateral speed, yaw and yaw rate.

**Table 2:** List of selected features from each signal. # represent features.

Data	# Extracted Features	# Selected Features	Features
EEG	270	11	FP1: β, FP2: θ, (θ+α)/(α+β)
			FP2: θ, (θ+α)/(α+β)
			FPz: β, θ/β, (θ+α)/β
			F4: θ
			F7: θ
			FC2: θ/β, α/β
EOG	9	5	Start position of blink, blink duration calculated from the start position of blink to the end value of blink, PERCLOS, blink rate, blink count.
ECG	14	9	**Time:** sdHR, SDNN, NN50, pNN50.
			**Frequency:** LF, HF, LF/HF ratio.
			**Non-linear:** dfaAlpha, SampEn.
GSR	10	4	**Time:** The amplitude of the peaks, duration of the rise time of each peak, mean value.
			**Frequency:** Average power of the signal under 1 Hz.
RR	9	7	**Time:** Mean value, standard deviation, kurtosis.
			**Frequency**: Power spectra power between the frequency ranges [0, 0.1], [0.2, 0.3], [0.4, 0.7], and [0.7, 1].
Vehicular data	11	6	Standard deviation of lateral speed.
			Standard deviation of lateral speed yaw.
			Steering wheel entropy, high frequency component (0.3 Hz), and number of zero crossings.
			Lanex or fraction of lane exit from lane departure.
All	323	42	Best subset of features after feature selection.

**Table 3:** Confusion matrix of k-nearest neighbour (k-NN), support vector machine (SVM), and random forest (RF) multiclass classification on the test dataset. The grey cells represent the true positive (TP) value. TP represents the number of observations that were correctly classified, and the precision value in percentage.

Predicted Class	Actual Class								
	k-NN			SVM			RF		
	Baseline	1-back	2-back	Baseline	1-back	2-back	Baseline	1-back	2-back
Baseline	60 (65%)	18 (20%)	14 (15%)	66 (72%)	13 (14%)	13 (14%)	70 (76%)	15 (16%)	7 (8%)
1-back	24 (34%)	39 (56%)	7 (10%)	22 (31%)	39 (56%)	9 (13%)	24 (34%)	36 (51%)	10 (14%)
2-back	13 (25%)	19 (35%)	21 (40%)	16 (30%)	12 (23%)	25 (47%)	13 (25%)	8 (15%)	32 (60%)

**Table 4:** Classification summary of multiclass classification for k-NN, SVM, and RF on the test dataset. Where the target classes are baseline (BL) or no-task, 1-back, and 2-back task. SEN: Sensitivity; SPE: Specificity; PRE: Precision; ACC: Accuracy; and BACC: Balanced accuracy.

Criteria	k-NN			SVM			RF		
	BL	1-back	2-back	BL	1-back	2-back	BL	1-back	2-back
TP	60	39	21	66	39	25	70	36	32
FP	32	31	32	26	31	28	22	34	21
FN	37	37	21	38	25	22	37	23	17
TN	86	108	141	85	120	140	86	122	145
PRE	0.65	0.56	0.40	0.72	0.56	0.47	0.76	0.51	0.60
SEN	0.62	0.51	0.50	0.63	0.61	0.53	0.65	0.61	0.65
SPE	0.73	0.78	0.82	0.77	0.79	0.83	0.80	0.78	0.87
BACC	0.68	0.65	0.63	0.70	0.69	0.67	0.73	0.68	0.75
*F*_1_-score	0.63	0.53	0.13	0.67	0.58	0.50	0.70	0.56	0.63
MCC	0.35	0.30	0.44	0.40	0.39	0.35	0.46	0.37	0.51

**Table 5:** Performance summary of the classifiers for binary classification on the test dataset.

Criteria	*BSet-1*			*BSet-2*		
	k-NN	SVM	RF	k-NN	SVM	RF
Task group (P)	52	52	52	126	126	126
Baseline group (N)	163	163	163	89	89	89
TP	20	27	38	116	104	107
FP	8	10	11	47	43	36
FN	32	25	14	10	22	19
TN	155	153	152	42	46	53
Sensitivity	0.38	0.52	0.78	0.92	0.83	0.84
Specificity	0.95	0.86	0.91	0.47	0.52	0.60
Accuracy	0.81	0.84	0.88	0.73	0.70	0.74
BACC	0.67	0.73	0.85	0.70	0.67	0.72
*F*_1_-score	0.50	0.61	0.75	0.80	0.76	0.80
MCC	0.43	0.52	0.68	0.45	0.36	0.46
